# Phytohormones Signaling Pathways and ROS Involvement in Seed Germination

**DOI:** 10.3389/fpls.2016.00864

**Published:** 2016-06-15

**Authors:** Krystyna Oracz, Stanisław Karpiński

**Affiliations:** ^1^Department of Plant Physiology, Warsaw University of Life SciencesWarsaw, Poland; ^2^Department of Plant Genetics, Breeding and Biotechnology, Warsaw University of Life SciencesWarsaw, Poland

**Keywords:** abiotic stress response, molecular regulators, phytohormones, PCD, ROS, seeds germination

## Abstract

Phytohormones and reactive oxygen species (ROS) are major determinants of the regulation of development and stress responses in plants. During life cycle of these organisms, signaling networks of plant growth regulators and ROS interact in order to render an appropriate developmental and environmental response. In plant’s photosynthetic (e.g., leaves) and non-photosynthetic (e.g., seeds) tissues, enhanced and suboptimal ROS production is usually associated with stress, which in extreme cases can be lethal to cells, a whole organ or even an organism. However, controlled production of ROS is appreciated for cellular signaling. Despite the current progress that has been made in plant biology and increasing number of findings that have revealed roles of ROS and hormonal signaling in germination, some questions still arise, e.g., what are the downstream protein targets modified by ROS enabling stimulus-specific cellular responses of the seed? Or which molecular regulators allow ROS/phytohormones interactions and what is their function in seed life? In this particular review the role of some transcription factors, kinases and phosphatases is discussed, especially those which usually known to be involved in ROS and hormonal signal transduction under stress in plants, may also play a role in the regulation of processes occurring in seeds. The summarized recent findings regarding particular ROS- and phytohormones-related regulatory proteins, as well as their integration, allowed to propose a novel, possible model of action of LESION SIMULATING DISEASE 1, ENHANCED DISEASE SUSCEPTIBILITY 1, and PHYTOALEXIN DEFICIENT 4 functioning during seeds life.

## Introduction

The complexity of regulatory tools operating in each living cell endows plants and seeds with the ability to respond, acclimatize and survive in a variable natural environment. The embryo contained within the seed is a sporophytic stage of plant generation and the switch from embryonic to germinative growth is the first critical phase of the transition in the plant life cycle. The germination of seeds of most higher plants species, including *Arabidopsis thaliana*, ends with the visible protrusion of covering layers (i.e., testa and endosperm) by an elongated radicle. Depending on the interaction between the weakened endosperm surrounding the radicle and on factors controlling embryo growth potential [i.e., phytohormones and reactive oxygen species (ROS)], proper seedling establishment either occurs or not (Oracz et al., 2012; Voegele et al., 2012). The failure of an intact viable seed to complete germination under optimal conditions is defined by dormancy. This phenomenon is an adaptive trait improving the survival of plant species in a nature by optimizing the distribution of germination over a period of time. The alleviation of seed dormancy is controlled by environmental factors (e.g., light and temperature) and endogenous cues (e.g., ROS and phytohormones).

The role of abscisic acid (ABA) in dormancy onset during seed development is well documented by genetic and physiological studies. A dynamic balance in the metabolism of ABA and its antagonists – gibberellins (GA), as well as their signaling pathways interacting with ROS-induced signals determines whether an imbibed seed will complete germination or remain dormant. Another phytohormone counteracting ABA effect is ethylene (ET). Studies using inhibitors of ET biosynthesis or action indicated the involvement of this phytohormone in the regulation of germination and dormancy *via* crosstalk with ABA, GA, ROS, and cyanide (Oracz et al., 2008, 2009). Although salicylic acid (SA) is not essential for germination under optimal growth conditions, the genetic studies using *A. thaliana* mutants such as: *gasa4* (*gibberellin-regulated protein 4*), *ein2-2* (*ethylene insensitive 2-2*), *aao3-4* (*abscisic-aldehyde oxidase 3-4*), and *sid2* (*SA induction deficient*) indicated that the mechanism of action of SA in various developmental processes of plants, involved its interactions with other phytohormones (i.e., GA, ABA, and ET), as well as with ROS (Lee et al., 2010; Wituszyńska et al., 2013).

**FIGURE 1:**
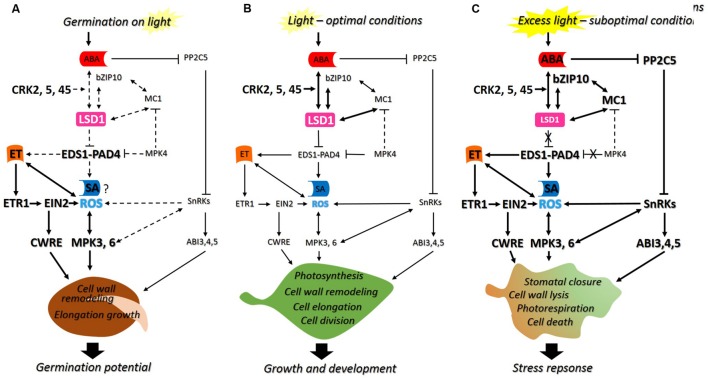
**The proposed model of action of LESION SIMULATING DISEASE 1 (LSD1), ENHANCED DISEASE SUSCEPTIBILITY 1–PHYTOALEXIN DEFICIENT 4 (EDS1–PAD4) and other molecular regulators: (A) during seed germination in light, as well as in foliar tissues exposed to (B) optimal or (C) suboptimal conditions.** LSD1 is a negative regulator of cell death and suppresses EDS1–PAD4 in laboratory conditions. This regulatory hub integrates signals induced by reactive oxygen species (ROS) and phytohormones [abscisic acid (ABA), ethylene (ET), and salicylic acid (SA)] during plants growth and development. It is highlighted that the mechanism of action of LSD1 and EDS1–PAD4 is conditionally regulated. In seeds imbibing in the presence of light, LSD1 suppresses EDS1–PAD4, thus it prevents cell death and promotes cell wall remodeling required for cell elongation of radicle during germination **(A)**. In plants growing in optimal conditions the inhibition of EDS1–PAD4 by LSD1 results in positive modulation of photosynthesis, cell wall remodeling, elongation, and division of cells crucial for proper growth and development **(B)**. The perturbations in photosynthetic tissue caused by suboptimal conditions lead to limitations of LSD1 suppression effect on EDS1–PAD4, resulting in induction of stress response and acclimation achieved by modulation of stomatal closure, photorespiration, cell wall lysis and cell death **(C)**. The dotted lines indicate possible interactions (not yet confirmed). The size of letters and thickness of arrows reflects to the effect (increase/decrease) of particular interactions between regulators.

Despite the current progress that has been made in plant science and cellular signaling, there is still far from complete understanding of biological functions of several regulatory proteins involved in signaling network of plant growth regulators and ROS occurring in non-photosynthetic (seeds) and photosynthetic (foliar) cells. Hence this review presents: (i) shortly summarized role of ROS and phytohormones in seeds and (ii) discussed novel findings about particular molecular regulators of ROS and phytohormone signaling with previously defined roles in plants acclimation and defense, which can also be involved in the regulation of germination. In addition, possible models of action of regulatory proteins integrating signals induced by ROS and phytohormones [e.g., LESION SIMULATING DISEASE 1/ENHANCED DISEASE SUSCEPTIBILITY 1/PHYTOALEXIN DEFICIENT 4 (LSD1/EDS1/PAD4)] in germinating seeds and plants exposed to optimal and/or suboptimal conditions are presented (**Figure 1**).

## ROS and Phytohormones Interactions in Seeds

The ability of ROS to interact with all cellular substances makes them important regulators of plant growth and development. They refer to a diverse group of reactive molecules such as: (i) superoxide ($O_{2}^{•-}$) or hydroxyl (^•^OH) radicals and singlet oxygen (^1^O_2_), that have a very short half-life-time and can be sensed at the production site, as well as 2) relatively stable with longer life-span hydrogen peroxide (H_2_O_2_) that can diffuse within and between cells and can be sensed remotely (Foyer and Noctor, 2012; Karpiński et al., 2013). In seeds, ROS are produced from embryogenesis to germination, i.e., in metabolically active cells, but also in quiescent dry tissues during after-ripening and storage, owing to various mechanisms depending on seed moisture content (Basbouss-Serhal et al., 2016). Although progress has been made in cellular ROS signaling, plant cells possess still unidentified specific ROS sensors that process and translate this information into respective molecular output programs (Mittler et al., 2011; Foyer and Noctor, 2012; Karpiński et al., 2013). During imbibition, embryo cells driven by water uptake elongate prior to the completion of germination, what is required for radicle protrusion. This type of cell growth is controlled by the cell wall (apoplast) and loosening of this structure is required. In addition to the direct mechanism of apoplastic ROS in the scission of cell wall polysaccharides, cell wall remodeling proteins (CWRP) provide an additional mechanism leading to cell wall loosening and cell extension during germination and seedling growth (Morris et al., 2011; Scheler et al., 2015).

The existence of crosstalk between ROS and hormonal signaling pathways in seeds, mediating processes such as: dormancy release, after-ripening and germination, was suggested (Oracz et al., 2008, 2009; Bahin et al., 2011). The accumulation of apoplastic ROS in a radicle and endosperm is developmentally and hormonally regulated. It was demonstrated that it may be inhibited by ABA thus resulting in the inhibition of endosperm rupture, while GA counteracting the ABA effect in the radicle stimulate germination of *Lepidium sativum* seeds (Graeber et al., 2010). The results of Bahin et al. (2011) showed that ROS-mediated dormancy alleviation in *Hordeum vulgare* seeds relied on the regulation of expression of genes encoding proteins involved in GA metabolism (*GA2ox3 – GIBBERELLIN 2 OXIDASE 3*, *GA20ox1 – GIBBERELLIN 20 OXIDASE 1*) and of GA-inducible genes (*ExpA11, EXPANSIN A11*). Interestingly, authors also suggested that changes in this type of seeds triggered by ROS did not engage the interaction with ABA signaling, even if a small increase in ABA content was observed. Moreover, it is known that in cereals ROS play a role in phytohormone-regulated programmed cell death (PCD) of aleurone cells. This process is stimulated by GA which induces ROS accumulation, whereas ABA maintains low ROS concentrations through activation of the alternative oxidase pathway and the ROS-scavenging systems (Bethke et al., 2007). The examples of spatial-temporal patterns of PCD occurring in tissues of plants under stress conditions and germinating seeds, obviously emphasize the role of ROS and phytohormones in regulation of these processes. However, the role of molecular regulators involved in ROS/hormonal induction and regulation of PCD, such as LSD1, EDS1, and PAD4 during germination is very elusive. The novel findings about LSD1/EDS1/PAD4 and other regulators of ROS/phytohormones signaling during stress response in plants (e.g., kinases and phosphatases) which are also involved in the regulation of processes occurring in seeds is discussed below.

## LSD1/EDS1/PAD4 – A Regulatory Hub of Germination?

The intrinsic genetic program operating in plant cells comprise a combination of mechanisms of action of various molecular regulators involved in signaling pathways induced by environmental and endogenous signals. The example of gene encoding regulatory protein integrating signals induced by ROS and phytohormones in response to diverse stresses is *LSD1* (Mühlenbock et al., 2008; Karpiński et al., 2013; Szechyńska-Hebda et al., 2016). The *lsd1* belongs to one of the best characterized *A. thaliana* mutants in the context of deregulated cell death. It is important to note that *lsd1* traits depend on *EDS1* and its interacting partner *PAD4*. The *A. thaliana* EDS1 and PAD4 constitute a regulatory hub for gene-mediated and basal resistance. Both regulators are required for accumulation of ROS, ET and SA in plants exposed to suboptimal conditions. Null mutations in *EDS1* and *PAD4* revert the *lsd1*-related limitation of foliar gas exchange, accumulation of mentioned plant growth regulators, and subsequent PCD (Mühlenbock et al., 2008; Karpiński et al., 2013; Szechyńska-Hebda et al., 2016). Moreover, Wituszyńska et al. (2013) demonstrated that features of *lsd1* depend on exogenous conditions. These authors have shown that *lsd1* is weaker and less tolerant in ambient laboratory growth conditions, but is more tolerant to combined drought and high light stress than wild type (WT) plants. Moreover, in the natural field WT and *lsd1* performed similarly. Such specific conditional differences were also reflected at gene expression levels. Based on that results it was suggested that LSD1/EDS1/PAD4 constitute a regulatory hub of transcription factors integrating photosynthesis, water use efficiency, ROS/hormonal cellular homeostasis with seed yield during plant growth and development in the field (Wituszyńska et al., 2013).

In spite of the fact that LSD1 and EDS1 are expected to regulate many cellular processes in plants, the knowledge about their function in seed physiology is poor. The recent data presented by Łkeczycka et al. (2014) implied that non-dormant *lsd1* seeds of *A. thaliana* mutants germinated more slowly than WT and *eds1* while placed in darkness, and the exposure to constant light did not significantly improve *lsd1* germination. It was observed that in comparison to *lsd1*, seeds of *eds1* mutant placed in light conditions shown similar pattern to well germinating WT but they were slower in darkness. Authors postulated that LSD1 plays a determinative role in the regulation of seed germination by suppression of EDS1-dependent PCD, and that it may act in a light-dependent manner (Łkeczycka et al., 2014). In another study it was shown that the activation of EDS1 and PAD4 was not only dependent on LSD1 suppression, but also on MPK4 since double *mpk4/eds1* and *mpk4/pad4* mutants have partially reverted the *mpk4* dwarfed phenotype (Petersen et al., 2010). Taking into account that LSD1 is a negative, conditional regulator of PCD, acting as ROS and hormonal rheostat preventing the pro-death pathway below certain ROS levels, it may happen that in *lsd1* seeds uncontrolled production of ROS causes loss of seed viability, which then results in a decreased percentage of germination. Interestingly, this conclusion taken on the basis of results obtained on seeds is supported by data published by other authors showing that LSD1 inhibits EDS1-mediated cell death in plants exposed to excess light (Mühlenbock et al., 2008; Wituszyńska et al., 2013, 2015). The LSD1 contains three zinc finger domains that have been demonstrated to be responsible for interaction with many proteins, including metacaspase MC1 and transcription factor bZIP10 (Coll et al., 2010, 2011). Moreover, LSD1 has been postulated as being a transcription factor by itself (Wituszyńska et al., 2013). Hence, it may regulate an expression of a broad spectrum of genes involved in different signaling and metabolic pathways. This assumption is supported by the transcriptome data presented in Wituszyńska et al. (2013), where genes involved in: (i) synthesis of germination inhibitors, such as ABA and SA, (ii) redox status regulation, (iii) cell wall remodeling, and (iv) development, were significantly deregulated in the *lsd1* null mutant in both: laboratory and field conditions. This pattern of gene expression observed in *lsd1* is similar to that presented in the study of Morris et al. (2011), where analogous groups of genes were expressed in a tissue-specific manner during endosperm weakening of germinating *L. sativum* seeds in response to phytohormones. Searching for the explanation of the possible mechanism of LSD1 action it was postulated that in *lsd1* the transcription of many genes was altered due to the secondary effect of ROS and hormonal imbalance as well as due to perturbations in cell wall remodeling, thus resulting in decreased rates of germination (Łkeczycka et al., 2014).

Taking into consideration an up-to-date knowledge about functions of PCD-related regulators in seeds and plants the novel, possible model of its action is proposed in this review and presented on the **Figure 1**. There are summarized the latest findings about the role of LSD1/EDS1/PAD4 in a signaling network of phytohormones (ABA, SA, and ET) and ROS operating in: non-photosynthetic (seed) (**Figure 1A**) and photosynthetic tissues (plant) (**Figures 1B,C**), exposed to optimal (**Figures 1A,B**) and/or suboptimal conditions (**Figure 1C**). It is emphasized that the same regulatory proteins may act differentially, in the manner dependent on the type of tissue as well as environmental conditions. Hence, it is highlighted that the mechanism of LSD1 and EDS1–PAD4 action as molecular regulators is conditional (**Figure 1**).

## Kinases and Phosphatases as Molecular Regulators of Seed-Related Events

Signal transduction and basic cellular processes associated with the regulation of cellular homeostasis are regulated *via* protein phosphorylation and de-phosphorylation in response to different stimuli. The various evidences confirm that mitogen-activated protein kinases (MAPK) and phosphatases are important regulators of these processes. The increasing number of evidences emphasize the role of both types of enzymes in directing cellular responses to a diverse array of stimuli, especially in the context of the response of photosynthetic tissue to stress conditions. Interestingly, the last decade brought discoveries indicating that MAPK also play a role in modulation of processes occurring in non-photosynthetic plant organs such as seeds. The MAPK are serine/threonine kinases that can phosphorylate a wide range of protein targets, including transcription factors and/or other kinases. It was shown that phosphorylation of the MYB44 transcription factor by MPK3 and MPK6 (MITOGEN ACTIVATED PROTEIN KINASE 3 and 6) was necessary for its function as a negative regulator of GA- and ABA-mediated control of seed germination (Nguyen et al., 2012). The data presented by Liu et al. (2011) indicated that *Glycine max* MPK4 functioned similarly to *A. thaliana* MPK4 (MITOGEN ACTIVATED PROTEIN KINASE 4). It is interesting that *A. thaliana mpk4* null mutant is semi-lethal and does not produce seeds. Other lines of evidences obtained in biochemical and genetic studies have indicated that protein post-translational modifications (i.e., phosphorylation and oxidation) play a role in the regulation of the physiological status of the cell, gene expression and seed germination (Oracz et al., 2007; Lounifi et al., 2013). The role of mRNA oxidation of *MPK1* (*MITOGEN ACTIVATED PROTEIN KINASE 1*) in the release of *Helianthus annuus* embryo dormancy was reported (Bazin et al., 2011). It was also shown that expression of *MPK6* and some other genes related to ROS signaling (e.g., *Ser/ThrPK* – *Ser/Thr PROTEIN KINASE*, *PTP* – *PROTEIN Tyr PHOSPHATASE*) was differentially affected by dormancy alleviation either during after-ripening or by cyanide treatment in *H. annuus* embryos (Oracz et al., 2009). These authors concluded that the effect of cyanide on gene expression was likely to be mediated by ROS. Brock et al. (2010) showed that the PP2C5 (PROTEIN PHOSPHATASE 2C 5) co-localized and interacted with stress-induced MPK3, MPK4, and MPK6 predominantly in the nucleus and thus suggested that PP2C5 acted as a MAPK phosphatase positively regulating seed germination, stomatal closure and ABA-inducible genes expression. When seeds of the *pp2c5* mutant were sown on ABA-containing medium, the germination rates were significantly increased compared to WT seeds, thus indicating that the *pp2c5* mutant displayed partial insensitivity toward ABA (Brock et al., 2010).

The interesting group of regulatory proteins are cysteine-rich kinases (CRKs) which have been suggested to control various aspects of plant development, stress adaptation and are intricately linked to ROS/redox signaling (Wrzaczek et al., 2010; Foyer et al., 2012). The phenomics study performed by Bourdais et al. (2015) showed that despite the large number and high sequence conservation of this type of kinases, individual CRKs have intriguingly distinct functions in different aspects of plant life, including seed germination. These authors indicated that CRK2 and CRK5 play predominant roles in growth regulation and stress adaptation, respectively. While in other studies it was demonstrated that the knock-down of *CRK36* gene resulted in increased sensitivity to ABA and osmotic stress (Tanaka et al., 2012), and the *altered seed germination 6* (*asg6; crk2*) mutant has been associated with changes in seed germination in response to ABA (Bassel et al., 2011). Moreover, Zhang et al. (2012) indicated that CRK45 plays a role in the regulation of *A. thaliana* seed germination, early seedling development and abiotic stress responses by positively regulating ABA responses in these processes (**Figure 1**). Since ROS and phytohormones have been suggested to be involved in modulation of endosperm weakening and radicle growth during germination (Oracz et al., 2012; Voegele et al., 2012), the delayed endosperm rupture in *crk*s could be due to ROS and phytohormone imbalances in these mutants (Bourdais et al., 2015).

While discussing plant-specific kinases involved in plant response to abiotic stresses and ABA-dependent plant development, it is important to mention the SnRK2 family members. The SNF1-RELATED PROTEIN KINASE 2s (members of SnRK2), together with the PYRABACTIN RESISTANCE/PYRABACTIN-LIKE/REGULATORY COMPONENTS OF ABA RECEPTORS (PYR/PYL/RCAR), PP2C5 are core components of ABA signaling in plant photosynthetic tissues and in seeds. The redundant ABA-activated protein kinases such as: SRK2D/SnRK2.2, SRK2E/SnRK2.6/OST1, and SRK2I/SnRK2.3, were shown to control gene expression through the phosphorylation of ABI5 and the other transcription factors, such as AREB3 (ABA-RESPONSIVE ELEMENT BINDING PROTEIN 3), during seed development and dormancy (Nakashima et al., 2009) (**Figure 1**). The authors concluded that at least one of these kinases was necessary to protect seeds during desiccation and to maintain quiescent. Park et al. (2009) suggested that PP2Cs prevent phosphorylation and activation of SnRK2s and downstream factors. Since these three kinases affect the expression of PP2Cs, it is likely that fine-tuning of the phosphorylation status of SnRK2s, ABI5 and related transcription factors, such as AREB3, by PP2Cs might turn out to control their activities during seed development and germination.

Indicated herein examples of kinases and phosphatases the most often recognized as key molecular regulators of stress response, highlight their role also in the establishment of ROS and hormonal signaling homeostasis in seeds-related events.

## Conclusion

The findings presented and discussed above demonstrate that regulatory genes encoding various kinases, phosphatases and transcription factors with previously defined roles in the regulation of ROS and phytohormone signaling for PCD as well as abiotic stress responses are also involved in the regulation of seed germination. The roles and function of these protein regulators were almost exclusively defined in fully developed leaves, which are not representative of other organs, tissues or types of cells. These limitations have been recently addressed in several studies and indicated that plants evolved a highly complex ROS and hormonal signaling and regulatory system. However, the precise function and role of this system depends on plant organs, tissue or type of cells and plant developmental status as well as on environmental conditions. In this review it is discussed that LSD1 and EDS1–PAD4 which regulate immune defenses, light acclimation, photosynthesis and transpiration are also involved in the regulation of seed germination as summarized in **Figure 1**.

## Author Contributions

All authors listed, have made substantial, direct and intellectual contribution to the work, and approved it for publication.

## Conflict of Interest Statement

The authors declare that the research was conducted in the absence of any commercial or financial relationships that could be construed as a potential conflict of interest.

## References

[B1] Bahin E. Bailly C. Sotta B. Kranner I. Corbineau F. Leymarie J. (2011). Crosstalk between reactive oxygen species and hormonal signalling pathways regulates grain dormancy in barley. *Plant Cell Environ.* 34 980–993. 10.1111/j.1365-3040.2011.02298.x 21388415

[B2] Basbouss-Serhal I. Leymarie J. Bailly C. (2016). Fluctuation of *Arabidopsis* seed dormancy with relative humidity and temperature during dry storage. *J. Exp. Bot.* 67 119–130. 10.1093/jxb/erv439 26428064 PMC4682427

[B3] Bassel G. W. Glaab E. Marquez J. Holdsworth M. J. Bacardit J. (2011). Functional network construction in *Arabidopsis* using rule-based machine learning on large-scale data sets. *Plant Cell* 23 3101–3116. 10.1105/tpc.111.088153 21896882 PMC3203449

[B4] Bazin J. Langlade N. Vincourt P. Arribat S. Balzergue S. El-Maarouf-Bouteau H. (2011). Targeted mRNA oxidation regulates sunflower seed dormancy alleviation during dry after-ripening. *Plant Cell* 23 2196–2208. 10.1105/tpc.111.086694 21642546 PMC3160027

[B5] Bethke P. C. Libourel I. G. L. Aoyama N. Chung Y.-Y. Still D. W. Jones R. L. (2007). The *Arabidopsis* aleurone layer responds to nitric oxide, gibberellin, and abscisic acid and is sufficient and necessary for seed dormancy. *Plant Physiol.* 143 1173–1188. 10.1104/pp.106.093435 17220360 PMC1820924

[B6] Bourdais G. Burdiak P. Gauthier A. Nitsch L. Salojärvi J. Rayapuram C. (2015). Large-Scale phenomics identifies primary and fine-tuning roles for CRKs in responses related to oxidative stress. *PLoS Genet.* 11:e1005373 10.1371/journal.pgen.1005373 PMC4511522 26197346

[B7] Brock A. K. Willmann R. Kolb D. Grefen L. Lajunen H. M. Bethke G. (2010). The *Arabidopsis* mitogen-activated protein kinase phosphatase PP2C5 affects seed germination, stomatal aperture, and abscisic acid-inducible gene expression. *Plant Physiol.* 153 1098–1111. 10.1104/pp.110.156109 20488890 PMC2899920

[B8] Coll N. S. Epple P. Dangl J. L. (2011). Programmed cell death in the plant immune system. *Cell Death. Differ.* 18 1247–1256. 10.1038/cdd.2011.37 21475301 PMC3172094

[B9] Coll N. S. Vercammen D. Smidler A. Clover C. Van Breusegem F. Dangl J. L. (2010). *Arabidopsis* type I metacaspases control cell death. *Science* 330 1393–1397. 10.1126/science.1194980 21097903

[B10] Foyer C. H. Kerchev P. I. Hancock R. D. (2012). The ABA-INSENSITIVE-4 (ABI4) transcription factor links redox, hormone and sugar signaling pathways. *Plant Signal. Behav.* 7 276–281. 10.4161/psb.18770 22415048 PMC3404864

[B11] Foyer C. H. Noctor G. (2012). Managing the cellular redox hub in photosynthetic organisms. *Plant Cell Environ.* 35 199–201. 10.1111/j.1365-3040.2011.02453.x 22070467

[B12] Graeber K. Linkies A. Müller K. Wunchova A. Rott A. Leubner-Metzger G. (2010). Cross-species approaches to seed dormancy and germination: conservation and biodiversity of ABA-regulated mechanisms and the Brassicaceae DOG1 genes. *Plant Mol. Biol.* 73 67–87. 10.1007/s11103-009-9583-x 20013031

[B13] Karpiński S. Szechyńska-Hebda M. Wituszynska W. Burdiak P. (2013). Light acclimation, retrograde signalling, cell death and immune defences in plants. *Plant Cell Environ.* 36 736–744. 10.1111/pce.12018 23046215

[B14] Łkeczycka J. Karpiński S. Oracz K. (2014). “LESION SIMULATING DISEASE1 control germination of after-ripened *Arabidopsis* seeds by ROS metabolism and ABA signalling,” in *Proceedings of the Book of Abstracts 11th Conference of the International Society for Seed Science*, Changsha.

[B15] Lee S. Kim S. G. Park C. M. (2010). Salicylic acid promotes seed germination under high salinity by modulating antioxidant activity in *Arabidopsis*. *New Phytol.* 188 626–637. 10.1111/j.1469-8137.2010.03378.x 20663063

[B16] Liu J.-Z. Horstman H. D. Braun E. Graham M. A. Zhang C. Navarre D. (2011). Soybean homologs of MPK4 negatively regulate defense responses and positively regulate growth and development. *Plant Physiol.* 157 1363–1378. 10.1104/pp.111.185686 21878550 PMC3252160

[B17] Lounifi I. Arc E. Molassiotis A. Job D. Rajjou L. Tanou G. (2013). Interplay between protein carbonylation and nitrosylation in plants. *Proteomics* 13 568–578. 10.1002/pmic.201200304 23034931

[B18] Mittler R. Vanderauwera S. Suzuki N. Miller G. Tognetti V. B. Vandepoele K. (2011). ROS signaling: the new wave? *Trends Plant Sci.* 16 300–309. 10.1016/j.tplants.2011.03.007 21482172

[B19] Morris K. Linkies A. Müller K. Oracz K. Wang X. Lynn J. R. (2011). Regulation of seed germination in the close *Arabidopsis* relative *Lepidium sativum*: a global tissue specific transcript analysis. *Plant Physiol.* 155 1851–1870. 10.1104/pp.110.169706 21321254 PMC3091087

[B20] Mühlenbock P. Szechyńska-Hebda M. Plaszczyca M. Baudo M. Mateo A. Mullineaux P. M. (2008). Chloroplast signaling and LESION SIMULATING DISEASE 1 regulate crosstalk between light acclimation and immunity in *Arabidopsis*. *Plant Cell* 20 2339–2356. 10.1105/tpc.108.059618 18790826 PMC2570729

[B21] Nakashima K. Fujita Y. Kanamori N. Katagiri T. Umezawa T. Kidokoro S. (2009). Three *Arabidopsis* SnRK2 protein kinases, SRK2D/SnRK2.2, SRK2E/SnRK2.6/OST1 and SRK2I/SnRK2.3, involved in ABA signaling are essential for the control of seed development and dormancy. *Plant Cell Physiol.* 50 1345–1363. 10.1093/pcp/pcp083 19541597

[B22] Nguyen X. C. Hoang M. H. Kim H. S. Lee K. Liu X. M. Kim S. H. (2012). Phosphorylation of the transcriptional regulator MYB44 by mitogen activated protein kinase regulates *Arabidopsis* seed germination. *Biochem. Biophys. Res. Commun.* 423 703–708. 10.1016/j.bbrc.2012.06.019 22704933

[B23] Oracz K. El-Maarouf-Bouteau H. Bogatek R. Corbineau F. Bailly C. (2008). Release of sunflower seed dormancy by cyanide: crosstalk with ethylene signaling pathway. *J. Exp. Bot.* 59 2241–2251. 10.1093/jxb/ern089 18448476 PMC2413275

[B24] Oracz K. El-Maarouf Bouteau H. Farrant J. M. Cooper K. Belghazi M. Job C. (2007). ROS production and protein oxidation as a novel mechanism of seed dormancy alleviation. *Plant J.* 50 452–465. 10.1111/j.1365-313X.2007.03063.x 17376157

[B25] Oracz K. El-Maarouf-Bouteau H. Kranner I. Bogatek R. Corbineau F. Bailly C. (2009). The mechanisms involved in seed dormancy alleviation by hydrogen cyanide unravel the role of reactive oxygen species as key actors of cellular signalling during germination. *Plant Physiol.* 150 494–505. 10.1104/pp.109.138107 19329562 PMC2675718

[B26] Oracz K. Voegele A. Tarkowská D. Jacquemoud D. Turečková V. Urbanová T. (2012). Myrigalone A inhibits *Lepidium sativum* seed germination by interference with gibberellin metabolism and apoplastic superoxide production required for embryo extension growth and endosperm rupture. *Plant Cell Physiol.* 53 81–95. 10.1093/pcp/pcr124 21908442

[B27] Park S.-Y. Fung P. Nishimura N. (2009). Abscisic acid inhibits type 2C protein phosphatases via the PYR/PYL family of START proteins. *Science* 324 1068–1071. 10.1126/science.1173041 19407142 PMC2827199

[B28] Petersen K. Qiu J.-L. Lütje J. Fiil B. K. Hansen S. Mundy J. (2010). *Arabidopsis* MKS1 is involved in basal immunity and requires an intact N-terminal domain for proper function. *PLoS ONE* 5:e14364 10.1371/journal.pone.0014364 PMC3010986 21203436

[B29] Scheler C. Weitbrecht K. Pearce S. P. Hampstead A. Büttner-Mainik A. Lee K. J. (2015). Promotion of testa rupture during garden cress germination involves seed compartment-specific expression and activity of pectin methylesterases. *Plant Physiol.* 167 200–215. 10.1104/pp.114.247429 25429110 PMC4280999

[B30] Szechyńska-Hebda M. Czarnocka W. Hebda M. Karpiński S. (2016). PAD4, LSD1 and EDS1 regulate drought tolerance, plant biomass production, and cell wall properties. *Plant Cell Rep.* 35 527–539. 10.1007/s00299-015-1901-y 26754794

[B31] Tanaka H. Osakabe Y. Katsura S. Mizuno S. Maruyama K. Kusakabe K. (2012). Abiotic stress-inducible receptor-like kinases negatively control ABA signaling in *Arabidopsis*. *Plant J.* 70 599–613. 10.1111/j.1365-313X.2012.04901.x 22225700

[B32] Voegele A. Graeber K. Oracz K. Tarkowská D. Jacquemoud D. Turecková V. (2012). Embryo growth, testa permeability, and endosperm weakening are major targets for the environment ally regulated inhibition of *Lepidium sativum* seed germination by myrigalone A. *J. Exp. Bot.* 63 5337–5350. 10.1093/jxb/ers197 22821938 PMC3431005

[B33] Wituszyńska W. Ślesak I. Vanderauwera S. Szechyńska-Hebda M. Kornas A. Van Der Kelen K. (2013). LESION SIMULATING DISEASE1, ENHANCED DISEASE SUSCEPTIBILITY1, and PHYTOALEXIN DEFICIENT4 conditionally regulate cellular signaling homeostasis, photosynthesis, water use efficiency, and seed yield in *Arabidopsis*. *Plant Physiol.* 161 1795–1805. 10.1104/pp.112.208116 23400705 PMC3613456

[B34] Wituszyńska W. Szechyńska-Hebda M. Sobczak M. Rusaczonek A. Kozłowska-Makulska A. (2015). LESION SIMULATING DISEASE 1 and ENHANCED DISEASE SUSCEPTIBILITY 1 differentially regulate UV-C-induced photooxidative stress signalling and programmed cell death in *Arabidopsis thaliana*. *Plant Cell Environ.* 38 315530 10.1111/pce.12288 24471507

[B35] Wrzaczek M. Brosché M. Salojärvi J. Kangasjärvi S. Idänheimo N. Mersmann S. (2010). Transcriptional regulation of the CRK/DUF26 group of receptor-like protein kinases by ozone and plant hormones in *Arabidopsis*. *BMC Plant Biol.* 10:95 10.1186/1471-2229-10-95 PMC3095361 20500828

[B36] Zhang Z. Wu Y. Gao M. Zhang J. Kong Q. Liu Y. (2012). Disruption of PAMP-induced MAP kinase cascade by a *Pseudomonas syringae* effector activates plant immunity mediated by the NB-LRR protein SUMM2. *Cell Host Microbe* 11 253–263. 10.1016/j.chom.2012.01.015 22423965

